# ADHD diagnosis from multiple data sources with batch effects

**DOI:** 10.3389/fnsys.2012.00070

**Published:** 2012-10-08

**Authors:** Emanuele Olivetti, Susanne Greiner, Paolo Avesani

**Affiliations:** ^1^NeuroInformatics Laboratory, Bruno Kessler FoundationTrento, Italy; ^2^Center for Mind and Brain Sciences, University of TrentoTrento, Italy

**Keywords:** ADHD, batch effect, dissimilarity space, Bayesian hypothesis testing, multivariate pattern classification

## Abstract

The Attention Deficit Hyperactivity Disorder (ADHD) affects the school-age population and has large social costs. The scientific community is still lacking a pathophysiological model of the disorder and there are no objective biomarkers to support the diagnosis. In 2011 the ADHD-200 Consortium provided a rich, heterogeneous neuroimaging dataset aimed at studying neural correlates of ADHD and to promote the development of systems for automated diagnosis. Concurrently a competition was set up with the goal of addressing the wide range of different types of data for the accurate prediction of the presence of ADHD. Phenotypic information, structural magnetic resonance imaging (MRI) scans and resting state fMRI recordings were provided for nearly 1000 typical and non-typical young individuals. Data were collected by eight different research centers in the consortium. This work is not concerned with the main task of the contest, i.e., achieving a high prediction accuracy on the competition dataset, but we rather address the proper handling of such a heterogeneous dataset when performing classification-based analysis. Our interest lies in the clustered structure of the data causing the so-called *batch effects* which have strong impact when assessing the performance of classifiers built on the ADHD-200 dataset. We propose a method to eliminate the biases introduced by such batch effects. Its application on the ADHD-200 dataset generates such a significant drop in prediction accuracy that most of the conclusions from a standard analysis had to be revised. In addition we propose to adopt the dissimilarity representation to set up effective representation spaces for the heterogeneous ADHD-200 dataset. Moreover we propose to evaluate the quality of predictions through a recently proposed test of independence in order to cope with the unbalancedness of the dataset.

## 1. Introduction

The advance of computational methods for data analysis is opening new perspectives for exploiting of structural and functional magnetic resonance imaging (fMRI) data in the field of neuroscience. The statistical learning framework (Hastie et al., 2009), and specifically multivariate pattern analysis, is a prominent example of these methods. In this framework the approaches are data-driven, i.e., they do not require the complete and explicit modeling of the underlying physiology of the brain. For this reason these methods are referred to as *model free* or non-parametric.

The most intuitive application of multivariate pattern analysis to the domain of clinical studies is diagnosis. In diagnosis a sample of brain images is collected both from a population of typically developing subjects (controls) and from non-typically developing subjects (patients). A classification algorithm is trained on the data to produce a classifier that discriminates between patients and controls. The challenge is to achieve accurate prediction on future subjects. Since this approach is data-driven, a successful detection of the disease does not always correspond to a deeper understanding of the pathology. The classifier acts as an information extractor and the basic inference that is derived from an accurate classifier is that the data actually carry information about the condition of interest.

The adoption of this kind of approach for diagnosis has some drawbacks. Model free approaches are sensitive to the size of the training sample. The collection of a large amount of data, i.e., of a large number of controls and patients, is often a premise for a successful study based on multivariate pattern analysis.

In 2011 the ADHD-200 Initiative^1^ promoted the collection of a very large dataset about the Attention Defict Hyperactivity Disorder (ADHD) in the young population. Concurrently a related competition, called ADHD-200 Global Competition, was set up to foster the creation of automatic systems to diagnose ADHD. The motivation of the ADHD-200 Initiative was that, despite a large literature of empirical studies, the scientific community had not reached a comprehensive model of the disorder and the clinical community lacked objective biomarkers to support the diagnosis.

The main aspect of the ADHD-200 dataset is its size. It represents one of the major efforts in the area of publicly available neuroimaging datasets concerned with a specific aim. The large size of the dataset is structured along two lines: the number of subjects and the types of data available for each subject. The dataset includes nearly 1000 subjects divided among typically developing controls and patients with different levels of ADHD, i.e., *inattentive*, *hyperactive*, and *combined*. Each subject is described by a heterogeneous set of data such as structural MRI, resting state fMRI and phenotypical information.

Analyzing the heterogeneous ADHD-200 dataset for the goal of the competition generates many difficulties. First of all the different types of data have to be transformed into a homogeneous vectorial representation space because this is a requirement for the majority of the most popular classification algorithms (Hastie et al., 2009). Secondly, the aggregation of datasets recorded from different institutions generates a clustered structure within the data. One example is that the data from each institution create a cluster. The effect of this clustered structure is usually problematic during the analysis phase and goes under the name of *batch effect*. The presence of batch effects conflicts with one of the basic assumptions of the statistical learning theory, i.e., that the data are independent and identically distributed (*iid*). Non-iid data may lead to sub-optimal training. More importantly, non-iid data within the test set lead to biased estimates of the performance of the classifier. Last but not least, if the data of the test set are not independent from those of the training set then the estimated performance of the classifier becomes optimistically biased. Nevertheless the presence of a specific structure in the data can also be exploited to get a more accurate classification (Dundar et al., 2007). The third kind of difficulty in analyzing the ADHD-200 dataset is its unbalanced distribution of the subjects in the diagnostic groups. Table 1 reports that in the ADHD-200 dataset there are 575 typically developing subjects, 144 inattentive, 9 hyperactive, and 204 combined. In section 3 we will show that in unbalanced cases the standard statistic of prediction accuracy can be misleading to understand the actual performance of the classifier and that new methods should be adopted.

**Table 1:** **Subjects distribution of the ADHD-200 dataset with respect to the sites**.

**Site**	**0**	**1**	**2**	**3**	**All**
PKGU	146	67	2	30	245
BHBU	21	5	0	0	26
KKI	48	23	0	23	94
NIMP	26	8	0	14	48
NYU	132	72	2	47	253
OHSU	73	17	1	20	111
UPIT	89	2	0	6	97
WUSL	40	10	4	4	58
	575	204	9	144	932

The columns indicate the diagnostic group: 0 typically developing control, 1 ADHD combined, 2 ADHD hyperactive, and 3 ADHD inattentive. The acronyms of sites are as follows: PKGU, Peking University; BHBU, Bradley Hospital Brown University; KKI, Kennedy Krieger Institute; NIMP, Neuroimage Multi-Center Project; NYU, New York University; OHSU, Oregon Hospital Science University; UPIT, University of Pittsburgh; and WUSL, Washington University Saint Louis.

In this work we propose solutions for the three issues just described. We propose the use of the dissimilarity representation (Pekalska et al., 2002; Balcan et al., 2008; Chen et al., 2009) as a mean to construct a common representation space for all the heterogeneous types of data available in the ADHD-200 dataset. In the dissimilarity space representation the data from a given source, e.g., the structural MRI scan of a subject, are projected into a vector just by providing a source-specific distance function. Once the data of all sources of a subject are transformed into vectors, they can be concatenated into a larger vector that represents a homogeneous description of the subject over multiple data sources.

We propose to address the issue of the batch effect by following some of the ideas presented in Dundar et al. (2007). The structure of the ADHD-200 dataset presents the same two levels of batch effect modeled in Dundar et al. (2007), i.e., a site-level and a subject-level. The site-level is due to the site specificities in the data collection process. The subject-level is due to the availability of multiple fMRI recordings for some subjects. Besides implementing the solution presented in Dundar et al. (2007), which is tailored to the improvement of the classification accuracy, we propose to conduct three different estimation processes of the classification performance in order to avoid the potential biases explained above.

The third issue, i.e., assessing the performance of the classifier on the unbalanced ADHD-200 dataset, is addressed by testing the statistical dependence between the predictions and the actual diagnostic group of each subject. We draw from the statistics literature and adopt a recent Bayesian test of independence for contingency tables (Casella and Moreno, 2009; Olivetti et al., 2012a).

The structure of this paper is the following. In section 2 we describe the ADHD-200 dataset and part of the publicly available pre-processing pipelines from which we started our analysis. In section 3 we describe the three main ingredients of our work, i.e., the dissimilarity representation, the model of the batch effects and the test of independence to assess whether and how the proposed classification system was able to discriminate ADHD subjects from healthy subjects. In section 4 we illustrate the experiments we conducted with all the necessary details to implement the proposed methods. To conclude, in section 5 we discuss the results of our experiments and we show that the postulated batch effects are the major contribution in the apparently positive classification results.

## 2. Materials

Our study refers to the ADHD-200 initiative and dataset. The initiative is dedicated to support the scientific community in studying and understanding the neural basis of ADHD. The eight member institutions collected imaging datasets from almost 1000 young subjects (age 7–26) with and without ADHD, see Table 1 for details.

The diagnosis of ADHD was segmented in four different levels: typically developing, hyperactive, inattentive, and combined subjects. For each subject multiple types of data were collected: phenotypic data, structural (T1) MRI data, and fMRI resting-state data. For many subjects multiple fMRI resting state recordings were available. Accompanying phenotypic information included: age, gender, handedness, and IQ measure^2^. The ADHD-200 dataset is publicly available and freely distributed with the support of the International Neuroimaging Data-sharing Initiative^3^.

In 2011 the ADHD-200 initiative set up a global competition to develop diagnostic classification systems for ADHD diagnosis based on structural and fMRI data of the brain. Even though the ADHD-200 dataset comprised three different levels of the ADHD disorder and the healthy subjects, the competition was designed to discriminate only among three categories: typically developing, ADHD combined, and ADHD inattentive^4^. In this work we restrict our analysis to discriminating two diagnostic categories, i.e., controls and ADHD patients, by aggregating ADHD combined and inattentive patients into one class. This choice is motivated by the inherent difficulty of discriminating even just the two main categories. Moreover the aim of this work is eminently methodological and our claims are appropriately addressed even with such a simplifying restriction.

In 2011 the ADHD-200 dataset was delivered in two stages the first part to be considered as the train set for the construction of classifiers and the second part as test set for performance evaluation. In this work we consider only the aggregation of the two parts as a single dataset. We will discuss how we split it into train set and test set only in section 4 after the introduction of the batch effect model and the related performance estimation processes.

In the following we refer to the whole dataset comprising the data of 923 subjects where the diagnostic classes are distributed as follows: 62% typically developing control, 38% ADHD combined or inattentive. For a few subjects the structural (T1) magnetic resonance imaging or the resting state fMRI recording were not available or corrupted. These subjects were excluded from our study.

Some sites provided multiple recordings of resting state fMRI for many of their subjects. In our analysis we considered all the recordings in order to improve the training process without discarding or aggregating part of them. Table 2 shows the range of multiple resting state fMRI recordings with respect to the different sites. Different acquisition protocols were adopted by distinct sites. For example, in some sites the subjects were asked to keep their eyes closed while in other sites eyes were kept open. In this last case some sites proposed a fixation cross while others did not.

**Table 2:** **Details on resting state fMRI recordings in the ADHD-200 dataset with respect to the sites**.

**Site**	**PKGU**	**BHBU**	**KKI**	**NIMP**	**NYU**	**OHSU**	**UPIT**	**WUSL**
fMRI	1	1	1	1	1–2	1–4	1	1–6
Eyes	O	O	C	C	O	O	O	O
Screen	N	F	-	-	F	F	N	F

The first row shows the range of multiple recording sessions. The second row refers to the eye condition: O, open; C, closed. The third row shows the screen setup: F, fixation; N, no fixation.

For the initial preprocessing of neuroimaging data we refer to the Neuro Bureau initiative^5^ that provides high-quality and publicly available preprocessed versions of the ADHD-200 dataset in order to facilitate the development of algorithms for data analysis. Among the different pipelines supported by the Neuro Bureau initiative we focused on the data computed by the Burner and the Athena pipelines^6^. The Burner pipeline was managed by Carlton Chu using the Dartel toolbox from SPM8. The Athena pipeline was managed by Cameron Craddock using AFNI and FSL running on the Athena computer cluster at Virginia Tech's ARC.

The Burner pipeline created normalized gray matter maps. Structural images were segmented into gray matter and white matter probability maps. Then inter-subject registration was implemented through voxel-based morphometry (Ashburner and Friston, 2000) to the group average. The description of the pipeline reports this note which supports our investigation on batch effects: *“there are systematic biases in the segmented gray matters across different centers”*^7^.

The Athena pipeline is primarily focused on resting state fMRI data processing. The pre-processing of fMRI data included the registration into MNI space at 4 × 4 × 4 mm voxel resolution, slice time correction, a band pass filter between 0.009 and 0.08 Hz, and the removal of nuisance variance. For the analysis in the present study we focused on two alternative computational methods for encoding the information in fMRI signal: the former based on the notion of region of homogeneity (REHO), the latter based on spatial multiple regression for functional connectivity (SMR). Both of them are part of the Athena pipeline.

REHO (Zang et al., 2004) is a computational method that measures the similarity of the time series of a given voxel to those of its nearest neighbors. The Kendall's coefficient concordance is proposed as a measure of similarity. The output is a volume per subject where the value of each voxel is an estimate of the homogeneity of the BOLD signal during the resting state fMRI recording. We refer to these 10 volumes, which we denote as SMR0-9, when building the related representation space described in section 4.

The functional connectivity maps for the resting state network (SMR) were constructed using a modified approach based on dual regression proposed in Smith et al. (2009). A multiple regression was computed to extract the time courses of voxels corresponding to spatial templates of resting state network. The computation considered 10 distinct spatial templates. For each template the output is a volume where the value of a voxel is the correlation measure between the original and the extracted time courses. We refer to these volumes when building the representation spaces described in section 4.

In addition to all data reported above we considered also the information on head motion during the fMRI scan sessions. For each fMRI recording we referred to the motion parameters estimated during the computation of the head movement correction.

## 3. Methods

The proposed method consists of four main components: the construction of homogeneous representation spaces for all data sources, the batch effect model, a classification algorithm meant to extract information from the data and a statistical test to make inferences about the classification process. In this section we describe three of the four components while the actual classification algorithm and all the details necessary for implementation will be described in section 4. In the following we describe the dissimilarity representation technique together with the explicit model of the batch effects that we considered for building the representation spaces of all data sources. Then we introduce recently proposed a Bayesian test of independence between the predicted and the true class labels to assess whether the classifier built from the data is able to discriminate the two diagnostic groups.

### 3.1. The dissimilarity representation

The dissimilarity representation (Pekalska and Duin, 2005) is a Euclidean embedding technique, i.e., a method to represent general objects, e.g., structural (T1) MRI scans, as vectors. It is defined by first selecting a set of those objects, called *prototypes*, from their native space, e.g., a set of MRI scans from the available dataset. Then each new object, e.g., any new structural MRI scan, is mapped into the vector of distances from the prototypes. This representation (Pekalska et al., 2002; Balcan et al., 2008; Chen et al., 2009) is usually presented in the context of classification and clustering problems and was proven to keep the separation between classes when present in their original space (Balcan et al., 2006).

The dissimilarity representation is a *lossy* transformation in the sense that some information is lost when projecting the data into the dissimilarity space. In Pekalska et al. (2006) the approximation was studied to decide among competing prototype selection policies only for classification tasks. In Olivetti et al. (2012b) the approximation was characterized in the unsupervised setting and a scalable prototype selection policy was described.

Let X be the space of the objects of interest, e.g., structural (T1) MRI scans, and let X∈X. Let $d:X\times X\mapsto {\mathbb{R}}^{+}$ be a distance function between objects in X, e.g., the correlation distance. Note that *d* is not assumed to be necessarily metric. Let $\Pi =\{{\tilde{X}}_{1},\ldots ,{\tilde{X}}_{p}\}$, where $\forall i\;{\tilde{X}}_{i}\in X$ and *p* is finite. Each ${\tilde{X}}_{i}$ is called *prototype* or *landmark*. The *dissimilarity representation* or *projection*, is defined as $\phi_{\Pi }^{d}(X):X\mapsto {\mathbb{R}}^{p}$ s.t.

(1) $$\phi_{\Pi }^{d}(X)=[d(X,\;{\tilde{X}}_{1}),\;\ldots ,\;d(X,\;{\tilde{X}}_{p})]$$

and maps an object *X* from its original space X to a vector of ℝ^*p*^.

#### 3.1.1. Number and selection of the prototypes

The degree of approximation of the dissimilarity representation depends on the choice of the prototypes. In order to achieve a compact but accurate representation we need to define both the number of the prototypes and their selection process. First we illustrate a procedure to measure how accurate a given representation is. Then we describe the adopted procedure to select the prototypes. By using these two ingredients, in section 4 we will show how we selected the desired number of prototypes.

Following Olivetti et al. (2012b), we define the distance between projected objects as the Euclidean distance between them: Δ^*d*^_Π_(*X*, *X*′) = ||ϕ^*d*^_Π_(*X*) − ϕ^*d*^_Π_(*X*′) ||_2_, i.e., $\Delta_{\Pi }^{d}:X\times X\mapsto {\mathbb{R}}^{+}$. Other distances may be considered but we note that many learning algorithms rely on the Euclidean distance (Hastie et al., 2009) and for this reason we adopt it. It is intuitive that, in order to have an accurate dissimilarity representation, Δ^*d*^_Π_ and *d* must be strongly related. As a measure of the quality of approximation of the dissimilarity representation we adopt the Pearson correlation coefficient *r* between the two distances over all possible pairs of objects in the dataset. An accurate approximation of the relative distances between objects in X results in values of ρ far from zero and close to 1.

The definition of the set of prototypes with the goal of minimizing the loss of the dissimilarity projection is an open issue in the dissimilarity space representation literature. Following Pekalska et al. (2006) and Olivetti et al. (2012b), we adopt the *farthest first traversal* (FFT) selection algorithm, also known as *k*-center algorithm. This algorithm selects the prototypes sequentially: the first prototype is drawn at random from the dataset. Then any further prototype is defined as the point in the dataset maximizing the sum of the distances from the previously selected prototypes. This algorithm is both accurate and effective (Pekalska et al., 2006; Olivetti et al., 2012b).

### 3.2. Modelling the batch effects

The ADHD-200 dataset is the aggregation of datasets collected by multiple institutions. Moreover in the ADHD-200 dataset multiple fMRI resting state recordings are available for many of the subjects involved in the study. These facts motivate why at least two levels of batch effects should be expected. First each site is expected to have its own specificity about the collected MRI data and the specific sample of subjects selected for the study. MRI hardware specifications of each site, the actual MRI sequences used, the local ADHD and healthy population addressed and the local best practices at each step of the collection process are examples of the site specificity. A second-level of batch effect arises because in this work we consider each available run of fMRI recordings as a new example to be used for improving the classification step. This choice *virtually* increases the number of subjects from 923 to 1339. The availability of multiple recordings for some of the subjects creates a second-level of batch effect because the variability within subject is expected to be much lower than the variability across different subjects, even within the same site.

In data analysis the identification, modeling and removal of batch effects is mainly addressed by the literature in statistics and in the applied fields of epidemiology and genomics. To the best of our knowledge there is no neuroimaging literature on the issue of batch effects within data. In statistics and epidemiology the issue is also referred to as *correlated samples*, meaning that samples from the same batch share a certain degree of correlation not related to the phenomenon of interest of the study. Well known models to deal with correlated samples are the *random effect model* (Ishwaran, 2000) and the *generalized linear mixed effect model* (GLMM) (McCulloch et al., 2008). In genomics the batch effect literature was recently reviewed in Chen et al. (2011) where six algorithms for batch effect removal were compared. In this field the identification of batch effects is usually done by means of the principal variation component analysis (PVCA) algorithm (Boedigheimer et al., 2008). It is our understanding that the algorithms in the field of genomics are mainly devoted to removing the batch effects, while in the statistics literature there is more emphasis into modeling their effects. Moreover the techniques of the genomics literature are tightly related to the specificity of the genomic data, e.g., high variance in gene expression microarray data is usually related to a high degree of information, and not all the assumptions of that field of application might be transferred to the fields of neuroimaging and of disorder diagnosis in a straightforward way. In addition, the present work is concerned with statistical classification and we note that the issue of exploiting the batch effect structure within a dataset in classification problems has almost been neglected in the machine learning literature. To the best of our knowledge only in Dundar et al. (2007) a method was proposed to account for the two-level batch effect mentioned above, i.e., the site-level and the subject-level. The algorithm proposed in Dundar et al. (2007) is related to the GLMM algorithm, but while the GLMM is meant for explanatory data analysis, the algorithm we adopted is meant for predictive modeling, which is the aim of this paper.

The method of Dundar et al. (2007) is based on the simple idea of creating new binary variables, one for each site, where each variable indicates whether the given resting state run belongs to that site or not. Moreover even the second-level of the batch effect is modeled by additional binary variables, one for each subject, indicating to which subject the resting state run belongs to. The whole set of binary variables defines a binary vector where only two values at a time are set to 1 and all the other values are set to 0. This vector describes the two-level batch effect information for each available recording.

We notice that considering and modeling the batch effect structure has two potential effects on the analysis. First, we provide explicit information about natural structures present within the data. This may improve the classification performance when predictions are made from data belonging to the same batch. Second, we reduce the optimistic bias in the estimate of the performance of prediction if we account for these dependency structure when building the test set. It is straightforward to notice that if examples in the test set are not drawn independently from those of the train set, then the estimated performance of the classifier is optimistically biased. Moreover if the samples in the test set are not drawn independently *from each other*, then the estimated performance of the classifier is biased, even though not necessarily in an optimistic way.

### 3.3. Evaluation of the classification results

We used the dissimilarity representation to create a vectorial description of each subject of the ADHD-200 dataset for each available data source. A classification algorithm was then trained to assess the ADHD-related information in each data source. In a further group of experiments the vectors from multiple data sources of each subject were concatenated into a higher dimensional vector to extract information about the joint effect of multiple data sources. In all cases the evaluation of the classifier for discriminating among the classes of interest, i.e., controls and ADHD patients, was assessed through a statistical test.

**Figure 1:**
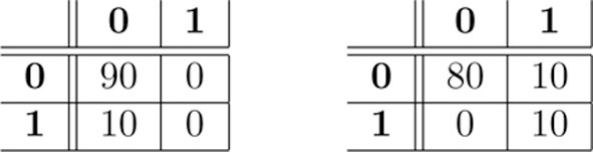
**Two examples of confusion matrices with true class labels on the rows and predicted class labels on the columns**. Both confusion matrices have the same estimated prediction accuracy, i.e., $\hat{P}A=\frac{90+0}{100}=\frac{80+10}{100}=0.1$. Nevertheless in the first case there is no evidence that the classifier is able to discriminate **0** from **1**, while in the second one there is.

As noted in Olivetti et al. (2012a), when the dataset is unbalanced with respect to the class-label distribution, the accuracy (or the error rate) of a classifier can be a misleading statistic to assess whether the classifier actually discriminated the classes. For example, given a test set of 100 instances where 90 are of class 0 and 10 of class 1, a classifier that incurs in 10 misclassification errors, i.e., the estimated error rate is $\hat{\epsilon }=10/100=0.1$, could be either highly accurate in discriminating the two classes or completely inaccurate. These two extreme cases are illustrated in Figure 1 by means of their confusion matrices. A confusion matrix reports the joint results of the predictions and the true class-labels. The table on the left shows a classifier that always predicts the most frequent class, i.e., class **0**, thus providing no evidence of learning the discrimination problem. Conversely, the table on the right shows evidence that the classifier correctly discriminates between the two classes and incurs in 10 errors over 90 examples but just for the frequent class. A solution to the issue of evaluating classifiers through the estimated accuracy in unbalanced cases is testing the full confusion matrix, as described in the next section.

#### 3.3.1. The bayesian test of independence

The literature answering the question “did the classifier learn to discriminate the classes?” was recently reviewed in Olivetti et al. (2012a) and a novel approach based on the analysis of the statistical independence between predicted and true class labels was proposed based on the work of Casella and Moreno (2009). In this work we adopt this latest approach that we summarize here. The intuitive idea is that, following the definition of statistical independence between random variables, in the case of a classifier predicting at random the predicted class labels are statistically independent from the true class labels. Conversely, the more the predictions match the true class labels, the stronger is the statistical dependence between them. The Bayesian test of independence between categorical variables first proposed in Casella and Moreno (2009) computes the ratio of the posterior distribution of the following two hypotheses:

- *H*_0_: the predictions are statistically *independent* from the true class labels.
- *H*_1_: the predictions are statistically *dependent* from the true class labels.

According to the Bayesian hypothesis testing framework (Kass and Raftery, 1995) that ratio can be rewritten as

(2) $$\frac{P(H_{1}|\text{data})}{P(H_{0}|\text{data})}=\frac{P(H_{1})}{P(H_{0})}\frac{P(\text{data}|H_{1})}{P(\text{data}|H_{0})}=\frac{P(H_{1})}{P(H_{0})}B_{10}$$

Where *B*_10_ is called *Bayes factor* and measures the evidence of the data in favor of *H*_1_ with respect to *H*_0_. When *B*_10_ ⋙ 1 the evidence in favor of *H*_1_ against *H*_0_ is strong. More detailed guidelines for the interpretation of these values are reported in Kass and Raftery (1995).

In order to compute *B*_10_ for the hypotheses of interest of this work it is necessary to define a sampling model for the confusion matrix under each hypothesis. Following Olivetti et al. (2012a) and Casella and Moreno (2009), we adopt the multinomial sampling model. A matter of debate of the computation of *B*_10_ is the definition of the prior distribution of the parameters under each hypothesis, namely *p*(θ_0_|H_0_) in *p*(data|θ_0_, H_0_) and *p*(θ_1_|H_1_) in *p*(data|θ_1_, H_1_). For this reason Casella and Moreno (2009) proposed the use of the *intrinsic prior* class (Berger and Pericchi, 1996) which concentrates the mass of *p*(θ_1_|H_1_) around *p*(θ_0_|H_0_) and creates multiple priors according to the observed data. Here we provide the approximate formula for *B*_10_ as derived in Casella and Moreno (2009)^8^:

(3) $$B_{10}(y,\;t)=\frac{(t+c^{2}-1)!}{(t+m+c^{2}-1)!}\left[\frac{\Gamma (m+c)\Gamma (m+c)}{\Gamma (t+c)\Gamma (t+c)}\right]\frac{1}{M}\sum_{k=1}^{M}\frac{\left(\prod r_{i}(x_{k})!)(\prod c_{j}(x_{k})!\right)}{\left(\prod r_{i}(y)!)(\prod c_{j}(y)!\right)}\times \frac{\prod \left(x_{k_{ij}}+y_{ij}\right)!}{\prod x_{k_{ij}}!}\frac{1}{\prod_{ij}{\hat{\theta }}_{ij}^{x_{k_{ij}}}}$$

where *y* is the confusion matrix, *m* = ∑_*ij*_y_*ij*_ is the size of the test set, **c** is the number of classes (**c** = 2 in our case), ${\hat{\theta }}_{ij}=\frac{y_{ij}+1}{m+c^{2}}$ and **x** = (x_*ij*_) ~ Multinomial(*t*, θ_11_, …, θ_*ab*_), *M* is the number of iterations of the sampling approximation, *r*_*i*_() and *c*_*j*_() are operators that return the *i*-th row and *j*-th column of a matrix and *t* is an integer parameter. Note that we define *B*_10_(*y*) = min_*t* ∈ {0 … *m*}_ *B*_10_(*y*, *t*).

## 4. Experiments

In this section we describe the detailed implementation of the methods illustrated in section 3 on the ADHD-200 dataset for the goal of discriminating typical from non-typical subjects. We first describe the pre-processing steps to create preliminary representations of the initial measurements, i.e., T1 structural MRI, REHO, and SMR volumes from resting state fMRI recordings, motion and phenotypic information, as described in section 2. Then we describe the distance function for each data source that was used to create the dissimilarity representation along with the selection of the number of prototypes. After the construction of the representation space we mention the classification algorithm that we used in this work and we describe the three train/testing processes that we designed to assess the importance of each data source for the prediction of the clinical group and the importance of the batch effects.

### 4.1. Preprocessing

Each structural (T1) MRI volume was first smoothed using a Gaussian filter with 3 voxels diameter and then downscaled such that each dimension was reduced by half. Then the transformed volume was flattened into a vector after removing the voxels outside the brain. A common brain mask was defined as the set of voxels for which at least 5% of the subjects had a non-zero value. Each resulting vector consisted in approximately 1.5 × 10^3^ values.

Each volume coming from the analysis of fMRI data through the Athena pipeline, i.e., REHO and SMR, was flattened into a vector after removing the voxels presenting no activity across 95% of the subjects. Each vector consisted in approximately 2.5 × 10^3^ values.

Following Dundar et al. (2007) and section 3, the batch effect information was encoded at two levels leading to a vector of binary variables. The first 10 binary variables corresponded to the 10 sites involved in the ADHD0-200 data collection process. The remaining 923 binary variables were one for each subject. For each measurement available in the dataset, e.g., MRI scan, resting state run, this binary random vector indicated the site and subject to which it belonged to by setting the corresponding site-variable and subject-variable to 1 and keeping all the remaining values at 0.

Phenotypic and motion data were *z*-scored.

### 4.2. Dissimilarity representation: source-specific distances and prototypes

For each data source we created the dissimilarity representation in order to have a compact representation. The distances adopted were:

- Distance among volumes (T1 MRI, REHO, and SMR): correlation distance, *d*(*a*, *b*) = 1 − *r*(*a*, *b*), where *r* is the Pearson correlation coefficient, of the related preprocessed vectors. This distance is the most common one in the neuroscience literature, see for example Kriegeskorte et al. (2008).
- Distance among vectors (phenotypic, motion, and batch effect): Euclidean distance.

The selection of the prototypes was done through the FFT algorithm described in section 3.1.1. We defined the number of prototypes by inspecting the correlation between the distances in the original space and the distances in the projected space. We observed high correlation, always *r*>0.85, with *p*=40 prototypes for all data sources. In all cases correlation reached the maximum value with such a number of prototypes or less. For this reason we selected that number of prototypes for all data sources.

### 4.3. Classification algorithm and testing schema

In this work we adopted an ensemble learning method based on model averaging and decision trees known as *extremely randomized trees* (Geurts et al., 2006). this classification algorithm is a variation of the popular random forest algorithm (Breiman, 2001). extremely randomized trees aim at reducing the variance of the resulting model. this class of algorithms is not influenced by different scaling in the data and it is known to fit both linear and non-linear aspects of the data.

In the following subsections we illustrate the classification experiments we conducted on the datasets resulting from the dissimilarity projection described above. The experiments aim at discriminating typical from non-typical developing subjects by estimating the classification performance. We conducted three groups of experiments with different cross-validation schemes to evaluate the importance of the batch effects. We describe the three groups of experiments and illustrate the classification results according to the common metric of the estimated classification accuracy, to the Bayesian test of independence introduced in section 3.1.1 and to the actual values of the confusion matrices.

All experiments were implemented in Python language on top of the numerical libraries NumPy and SciPy^9^, the machine learning library Scikit-Learn (Pedregosa et al., 2011)^10^ and the NiBabel library^11^ to access neuroimaging data formats.

#### 4.3.1. Information extraction from single source

In the first group of experiments a standard 10-fold stratified cross-validation scheme was used on each of the source-specific datasets. In this set of experiments subjects from the same site could appear in both the train and test sets of each cross-validation step. As mentioned in section 3.2 this fact should introduce optimistic bias if the batch effect at the site-level is strong. Moreover some subjects appeared multiple time in the dataset because multiple recordings were available for them. In this first group of experiments the same subject could appear both in the train set and the test set of each cross-validation step, causing the second-level of batch effect and the potential bias described in section 3.2. Table 3 presents the classification results for this first group of experiments, describing each data source in terms of log of the Bayes factor of *H*_1_ over *H*_0_, estimated accuracy, and the four values of the aggregated confusion matrix. The confusion matrix was computed by summing up the confusion matrices of each fold of the cross-validation.

**Table 3:** **Single source standard *k*-fold cross-validation**.

**Data**	**log(*B*_10_)**	**PA**	**TP**	**TN**	**FP**	**FN**
PHEN	10.17	0.61	178	651	170	340
STRU	31.19	0.66	112	776	45	406
MOT	−1.73	0.60	71	733	88	447
REHO	28.10	0.66	158	729	92	360
SMR0	3.95	0.61	90	737	84	428
SMR1	-0.66	0.60	78	731	90	440
SMR2	2.98	0.61	77	750	71	441
SMR3	1.72	0.61	80	741	80	438
SMR4	7.40	0.62	86	753	68	432
SMR5	0.46	0.60	77	739	82	441
SMR6	1.94	0.61	89	730	91	429
SMR7	4.50	0.61	110	713	108	408
SMR8	1.68	0.60	94	722	99	424
SMR9	3.95	0.61	90	737	84	428
BAEF	12.21	0.63	134	713	108	384

The table presents the results of the diagnosis from classifiers built upon a single type of data. The columns report the log of the Bayes factor for the hypothesis of dependence vs. independence [log(B_10_)], the prediction accuracy (PA), the number of true positive ADHD diagnosis (TP), the number of true negative ADHD diagnosis (TN), the number of false positive ADHD diagnosis (FP), and the number of false negative ADHD diagnosis (FN). The data considered in the analysis are: phenotypic data (PHEN), the structural MRI data (STRU), the motion parameters (MOT), the region of homogeneity of fMRI resting state (REHO), the spatial multiple regression of fMRI resting state (SMR0-9), and the batch effect (BAEF). Note that TP + TN + FP + FN = 1339 because multiple recordings are available for many subjects virtually increasing the total number of subjects.

In the second group of experiments the 10-fold stratified cross-validation scheme was modified such that the train/test sets split was done on the subjects. The aim was to remove part of the potential batch effect due to the presence of multiple recordings for some of the subjects. In each fold 1/10 of the subjects were drawn uniformly at random for the test set and the remaining 9/10 for the train set. In this way each subject would appear either in the train set or in the test set but not in both. Moreover we constrained the test set to have no more than one fMRI recording per subject by choosing one of them uniformly at random when necessary. Following this procedure we eliminated the possible bias due to non-independent examples within the test set. The results of classification for each data source are reported in Table 4. As expected the values of log(*B*_10_) were significantly reduced with respect to the values in Table 3, confirming the presence of the batch effect. We note that the accuracy values change marginally while the log(*B*_10_) values decrease significantly.

**Table 4:** **Single source 10-fold cross-validation on subjects**.

**Data**	**log(*B*_10_)**	**PA**	**TP**	**TN**	**FP**	**FN**
PHEN	−2.37	0.56	101	415	149	255
STRU	−0.97	0.61	42	520	44	314
MOT	−2.32	0.60	43	509	55	313
REHO	17.05	0.65	101	504	60	255
SMR0	1.64	0.61	68	497	67	288
SMR1	0.12	0.60	65	494	70	291
SMR2	−0.02	0.61	55	507	57	301
SMR3	−1.75	0.60	49	505	59	307
SMR4	−0.58	0.60	50	511	53	306
SMR5	−0.65	0.60	49	512	52	307
SMR6	−0.63	0.60	57	501	63	299
SMR7	−2.32	0.59	57	487	77	299
SMR8	5.21	0.62	73	503	61	283
SMR9	1.64	0.61	68	497	67	288
BAEF	10.37	0.64	99	489	75	257

The table reports the results of diagnosis for the classifiers built upon each single type of data. The columns report the log of the Bayes factor for the hypothesis of dependence vs. independence [log(B_10_)], the prediction accuracy (PA), the number of true positive ADHD diagnosis (TP), the number of true negative ADHD diagnosis (TN), the number of false positive ADHD diagnosis (FP), and the number of false negative ADHD diagnosis (FN). The data considered in the analysis are: phenotypic data (PHEN), the structural MRI data (STRU), the motion parameters (MOT), the region of homogeneity of fMRI resting state (REHO), the spatial multiple regression of fMRI resting state (SMR0-9), and the batch effect (BAEF).

The third group of experiments was meant to remove both levels of the postulated batch effect. We implemented leave-one-site-out cross-validation so that subjects from each site were either in the train or in the test set but not in both. Moreover this avoided the possibility of having recordings of the same subjects both in the train and test set. Additionally, as in the second group of experiments, we constrained the test set to have no more than one fMRI recording per subject by choosing one of them uniformly at random when necessary. Table 5 illustrates the single source results for this group of experiments. As expected most of the log(*B*_10_) values were significantly lower than those in Table 4 and Table 3 confirming the presence of the site batch effect.

**Table 5:** **Single source leave-one-site-out cross-validation**.

**Data**	**log(*B*_10_)**	**PA**	**TP**	**TN**	**FP**	**FN**
PHEN	−1.37	0.53	77	416	148	279
STRU	−0.87	0.56	29	494	70	327
MOT	1.41	0.55	29	482	82	327
REHO	−1.45	0.56	41	477	87	315
SMR0	−1.97	0.56	42	480	84	314
SMR1	−0.56	0.56	28	494	70	328
SMR2	−2.88	0.58	42	495	69	314
SMR3	−2.22	0.57	32	499	65	324
SMR4	−1.86	0.57	36	489	75	320
SMR5	−2.63	0.58	33	502	62	323
SMR6	−2.60	0.57	42	488	76	314
SMR7	−2.80	0.57	48	484	80	308
SMR8	−2.42	0.57	46	479	85	310
SMR9	−2.18	0.56	44	479	85	312
BAEF	3.10	0.50	82	376	188	274

The table shows the results of diagnosis from classifiers built upon each single type of data. The columns report the log of the Bayes factor for the hypothesis of dependence vs. independence [log(B_10_)], the prediction accuracy (PA), the number of true positive ADHD diagnosis (TP), the number of true negative ADHD diagnosis (TN), the number of false positive ADHD diagnosis (FP), and the number of false negative ADHD diagnosis (FN). The data considered in the analysis are: phenotypic data (PHEN), the structural MRI data (STRU), the motion parameters (MOT), the region of homogeneity of fMRI resting state (REHO), the spatial multiple regression of fMRI resting state (SMR0-9), and the batch effect (BAEF).

#### 4.3.2. Information extraction from multiple sources

In order to collect additional evidence of the presence of the batch effects within the data we conducted a further set of experiments combining multiple data sources. We set up the three experiments analogous to those described above for the single source case. This time we used two new datasets: one combining all data sources and one combining the four most informative data sources according to Table 3, i.e., phenotypic, structural, REHO, and batch effect. Table 6 confirms the importance of the batch effects showing the drastic decrease in the log(*B*_10_) and accuracy-level when the batch effects are gradually removed. The apparent positive results [80% accuracy and log(*B*_10_) > 100] of the first group of experiments, i.e., of basic 10-fold cross-validation, becomes null [50% accuracy and log(*B*_10_) ≈ 0] in the third group of results where the two-level batch effect is removed.

**Table 6:** **Multi source analysis**.

	**Data**	**log(*B*_10_)**	**PA**	**TP**	**TN**	**FP**	**FN**
10-fold CV	TOP4	147.78	0.80	351	719	102	167
	ALL	72.04	0.72	245	722	99	273
10-fold CV on subjects	TOP4	24.53	0.67	174	442	122	182
	ALL	22.57	0.67	140	476	88	216
Leave-one-site-out	TOP4	0.04	0.50	94	375	189	262
	ALL	−1.75	0.57	97	432	132	259

The table reports the results of diagnosis from classifiers built upon multiple sources of data. The table shows three different kind of analysis: standard 10-fold CV, 10-fold CV on subjects, and leave-one-site-out. The columns report log of the Bayes factor for the hypothesis of dependence vs. independence [log(B_10_)], the prediction accuracy (PA), the number of true positive ADHD diagnosis (TP), the number of true negative ADHD diagnosis (TN), the number of false positive ADHD diagnosis (FP), and the number of false negative ADHD diagnosis (FN). The data considered in the multi sources analysis are: all the available sources of data (ALL) and the four most predictive in single source analysis (TOP4), respectively phenotypic data, the structural MRI data, and the region of homogeneity of fMRI resting state and the batch effect.

## 5. Discussion

Table 3 reports the results of the investigation on single sources to recognize what kind of data is more informative and effective for the diagnosis of ADHD with the proposed classification method. The analysis of Table 3 does not consider the batch effects and the violation of the iid assumption. The estimate of prediction accuracy is computed by standard 10-fold cross-validation but only the Bayes factor clearly shows for which data source the classifier is learning the discrimination problem, i.e., phenotypic data (PHEN), structural data (STRU), regional homogeneity of fMRI resting state (REHO), and the batch effect data (BAEF). For these sources the log(*B*_10_) is above 10, which is considered (see Kass and Raftery, 1995) very strong evidence in support of *H*_1_ with respect to *H*_0_. The value of the prediction accuracy is instead less informative. For example, the prediction accuracy of 61% for phenotypic data is close to that of many other sources that are not significant, like SMR0, SMR2, SMR3, SMR6, SMR7, and SMR9.

The results presented in Table 4 show the effects of removing part of the postulated batch effects through the proposed method. The independence between train set and test set is kept at the subject-level by avoiding data from the same subject to appear in both of them. Moreover the presence of multiple records belonging to the same subject is avoided in the test set to eliminate the related bias. Both the prediction accuracy and the log(*B*_10_) drop with respect to the values of Table 3. While the change in accuracy is marginal (0–5%), the reduction of log(*B*_10_) is very strong^12^, which is evidence for the sensitivity of this parameter. These changes are clear evidence of the presence of the subject-level batch effect within the data.

In Table 5 all the postulated batch effects are removed by using the leave-one-site-out cross-validation scheme together with the constraint of one record per subject in the test set. The results show a drop in the values of prediction accuracy and log(*B*_10_) with respect to Table 4. Again the decrease in accuracy is marginal (0–5%) while the reduction of log(*B*_10_) is very strong. This fact is clear evidence in support of the presence of the batch effect at site-level, in addition to the one at subject-level. Moreover we observe that, for the single source analysis, no single data source is sufficient to support the hypothesis of discriminating controls from ADHD patients.

The joint analysis of multiple data sources is addressed in Table 6. The results show the impact of removing the batch effects in analogy to what was done for the single source analyses. In a broad sense the results in Table 6 also address the ADHD-200 Global Competition by using all available data sources for ADHD diagnosis. The analysis is conducted both for the whole set of data sources jointly and for the set of the four most informative data sources according to Table 3. The results show a significant drop in prediction accuracy and log(*B*_10_) when considering the batch effects in the estimation process. The prediction accuracy reaches an extreme value of 80 % when the batch effects are not taken into account, which drops to 50 % when all the batch effects are removed. The Bayes factor values show the same trend decreasing from log(*B*_10_) > 100 to log(*B*_10_) ≤ 0^13^.

## 6. Conclusions

Our results show that taking the batch effects into account and adopting a non-standard measure of the performance of the classifier, like the Bayesian test of independence, can prevent misleading conclusions in the analysis of large multi-site datasets. Nevertheless our results do not prove the absence of ADHD-related information within neuroimaging data. Our results are specific to the proposed representation spaces, i.e., of the dissimilarity representation, and of the proposed classification algorithm, i.e., extremely randomized trees. Different choices of the representation space and of the classification algorithm might lead to different results. What this work provides is a methodology to investigate the classification results in more detail.

In conclusion we argue that the assumptions on which the statistical learning framework relies may be violated by the presence of the batch effect and the consequence of these violations may lead to significant drawbacks during the analysis and may produce wrong inferences. In our study the estimated prediction accuracy decreased from 80 % to chance level by taking two levels of batch effect into account. Moreover the value of log(*B*_10_) for the batch effect encoded data can be used as an effective tool to detect when the batch effect structure may affect the inference. The very high values in the “BAEF” entry of Table 3 and of Table 4 are evidence of this.

We claim that the major challenges of having large datasets in the neuroscience domain, like the ADHD-200 dataset, are not just related to the inherent difficulties of data collection but they also involve the analysis and the interpretation of the results. This work provides some of the essential tools for moving toward the successful analysis of such datasets.

We speculate that the topic of batch effects in neuroimaging data analysis is not confined to site and subject levels but can extend to many other aspects of the neuroimaging data production cycle. Moreover many other approaches, different from the proposed one, should be attempted in order to deal with them. To the best of our knowledge this topic is lacking literature and we welcome future work in this direction.

### Conflict of interest statement

The authors declare that the research was conducted in the absence of any commercial or financial relationships that could be construed as a potential conflict of interest.

## Footnotes

^1^http://fcon_1000.projects.nitrc.org/indi/adhd200

^2^We did not consider other available phenotypic information because of the too many missing values.

^3^http://fcon_1000.projects.nitrc.org/indi/adhd200

^4^The *hyperactive* categories included only a few subjects.

^5^http://neurobureau.projects.nitrc.org/ADHD200/Introduction.html

^6^http://neurobureau.projects.nitrc.org/ADHD200/Data.html

^7^http://www.nitrc.org/plugins/mwiki/index.php?title=neurobureau:BurnerPipeline

^8^Here we take into account the corrections described in Olivetti et al. (2012a).

^9^http://www.scipy.org

^10^http://scikit-learn.org

^11^http://nipy.sourceforge.net/nibabel/

^12^Notice that this quantity is even in the log scale.

^13^According to Kass and Raftery (1995) when log(*B*_10_) ≤ 0 the evidence supports *H*_0_.
